# Expression of cytokine mRNA and protein in joints and lymphoid organs during the course of rat antigen-induced arthritis

**DOI:** 10.1186/ar1689

**Published:** 2005-02-17

**Authors:** Dirk Pohlers, Angela Siegling, Eberhard Buchner, Carsten B Schmidt-Weber, Ernesta Palombo-Kinne, Frank Emmrich, Rolf Bräuer, Raimund W Kinne

**Affiliations:** 1 Experimental Rheumatology Unit, Friedrich Schiller University Jena, Jena, Germany; 2 EUCODIS GmbH, Vienna, Austria; 3 Pfizer GmbH, Karlsruhe, Germany; 4 Swiss Institute for Asthma and Allergy Research (SIAF), Davos, Switzerland; 5 Institute of Clinical Immunology and Transfusion Medicine, University of Leipzig, Leipzig, Germany; 6 Institute of Pathology, Friedrich Schiller University Jena, Jena, Germany

## Abstract

Cytokine expression was assessed during antigen-induced arthritis (AIA) in synovial membrane (SM), inguinal lymph node (LN), and spleen using competitive RT-PCR and sandwich ELISA. In the SM, early elevations of IL-1β and IL-6 mRNA (by 6 hours; 450- and 200-fold, respectively) correlated with the joint swelling; a 6-fold increase in tumor necrosis factor α (TNFα) was not significant. Not only IL-2 and IFN-γ (which increased 10,000-fold and 200-fold, respectively), but also IL-5 and IL-10, increased acutely (6 hours – day 1; 3-fold and 35-fold, respectively) in the SM. In general, the protein levels in the SM for IL-1β, IL-6, TNFα, IFN-γ, IL-4, and IL-10 (increase from 4-fold to 15-fold) matched the course of mRNA expression. In the inguinal LN, there were early mRNA elevations of IL-6 (a 2.5-fold increase by 6 hours, which correlated positively with the joint swelling) and IL-2 (4-fold by 6 hours), as well as later rises of IL-4 and IL-5 (2.5- and 4-fold, respectively, by day 3). No significant elevations of the corresponding proteins in this tissue were observed, except for IL-1β (by day 6) and IL-10 (by day 1). In the spleen, there were significant mRNA elevations at 6 hours of IL-1β (1.5-fold), IL-6 (4-fold; positively correlated with the joint swelling), IFN-γ (3-fold), and IL-2 (7- to 10-fold). IL-5 and IL-10 (2- and 3-fold, respectively) peaked from 6 hours to day 3 in the spleen. Increases of the corresponding proteins were significant in comparison with day 0 only in the case of IL-2 (day 6). By day 6 (transition to the chronic phase), the mRNA for cytokines declined to or below prearthritis levels in all the tissues studied except for IL-1β in the SM and IL-6 in the spleen. AIA is thus characterized by four phenomena: early synovial activation of macrophages, T helper (Th)1-like, and Th2-like cells; late, well-segregated Th2-like responses in the inguinal LN; late, overlapping Th1-like/Th2-like peaks in the spleen; and chronic elevation of synovial IL-1β mRNA and spleen IL-6 mRNA.

## Introduction

CD4^+ ^T helper (Th) cells and macrophages infiltrate the synovial membrane (SM) during the course of rheumatoid arthritis (RA) [1-3]. Both cell types, when activated, appear to play a central role in promoting and maintaining the disease process [4,5], for example by producing certain sets of cytokines that influence the quality and extent of the inflammatory process [6]. Cytokines, in turn, can elicit the production of tissue-degrading enzymes, a mechanism eventually involved in tissue destruction and loss of articular function [5,7].

Many studies indicate that Th cells differentiate into functionally polarized effector subpopulations, producing either Th1- or Th2-like cytokines [8,9], although this concept has recently been re-evaluated [10] in a report that focused attention on the specific role and effects of individual cytokines. The Th1/Th2 subpopulations nevertheless appear differentially involved in several human and experimental immunological disorders, exerting either proinflammatory or regulatory functions [11]. Thus far, however, the evidence as to the expression of these cytokines in human RA is relatively limited and/or contradictory [12,13] and does not provide information on the time course and organ distribution of the cytokine profiles. Indeed, an extensive study of longitudinal cytokine profiles in RA is complicated by the influence of disease phases and/or treatments [14], most of which affect proportions and functions of lymphocytes and macrophages (reviewed in [1,2]). Experimental models of arthritis are therefore well suitable for learning about the sequence of cell activation in well-characterized phases of the disorders.

Antigen-induced arthritis (AIA) in the rat [15], a severe knee monoarticular arthritis induced by intra-articular administration of methylated bovine serum albumin (mBSA) after systemic immunization, is a suitable arthritis model inasmuch as CD4^+ ^T cells and macrophages infiltrate the SM [16] and its course consists of clearly discernible phases. The acute phase progresses within approximately 1 week into chronicity, that is, a status of low-grade inflammation with moderate joint destruction and bone formation [15]. Because of its prominently local character, AIA is a unique model for the analysis of cytokine patterns in locally driven immune responses, and also a useful counterpart for comparison with more systemic models of arthritis, such as adjuvant- and collagen-induced arthritis [17].

To elucidate the sequence and the interplay of cytokine gene activation in AIA, therefore, the mRNA expression of monokines and of Th1-like and Th2-like cytokines was analysed in the SM, regional lymph node (LN), and spleen of diseased rats by means of semiquantitative, competitive RT-PCR. To assess local translation into protein, the cytokine levels were also measured by ELISA. The analysis was carried out in mBSA-immunized animals before induction of disease (day 0), at 6 hours after injection of the arthritogen, and on days 1, 3, and 6 of the disease, the latter time point marking the transition to the chronic phase. For the sake of clarity, individual cytokines are assigned to monokine- and Th1-/Th2-like patterns, according to widely accepted schemes [8] and/or their prevalent cellular source in arthritis [2,18].

## Materials and methods

### Animals

#### Induction of arthritis

Female Lewis rats (140–190 g, age 7 to 10 weeks, Charles River Laboratories, Sulzfeld, Germany, or Animal Research Facility, Friedrich Schiller University) were immunized 21 and 14 days before induction of AIA with 2 ml (total volume) of a suspension containing 1 ml each of mBSA dissolved in PBS (500 μg/ml; Sigma, Deisenhofen, Germany), and complete Freund's adjuvant (2 mg/ml *Mycobacterium tuberculosis*; R37 Ra; Difco, Detroit, MI, USA) by multiple subcutaneous injections into both flanks of the animals. On day 0, AIA was induced by intra-articular injection of 100 μg mBSA in 50 μl of PBS into the right knee joint; the left knee received 50 μl of PBS and served as control. All animal studies were approved by the governmental commission for animal protection.

#### Scoring of arthritis

The disease course was monitored by repeated assessment of the bilateral swelling of the knee joint using a caliper (Kroeplin Längenmesstechnik, Schlüchtern, Germany). The swelling was expressed as the difference between the arthritic (right) and the control, unaffected joint (left).

### Assessment of cytokine mRNA expression

#### Tissue sampling and preparation

Samples of knee joint SM, inguinal LN, and spleen were obtained from rats killed at five time points: day 0, 6 hours, day 1, day 3, and day 6 of AIA (four to six rats per time point). After sacrifice, tissue pieces (approximately 2 to 5 mm^3^) were excised, snap-frozen in 500 μl guanidinium thiocyanate buffer, and kept at -70°C until processing.

#### Semiquantitative RT-PCR

Frozen tissues were homogenized, mRNA was isolated, cDNA was prepared and competitive RT-PCR was performed as previously described [19]. Quantification was not done until all cDNAs had been adjusted to equal β-actin mRNA content using semiquantitative RT-PCR with a competitor fragment, which contained the sequences corresponding to the primers [20]. From the present data, there was no experimental indication for the regulation of β-actin under arthritis conditions. In addition, the consistency between values found for mRNA (normalized to β-actin) and for protein (normalized to total protein) in the SM suggests little or no regulation of β-actin in this system. An amount of 2.5 × 10^-12 ^to 2.5 × 10^-19 ^moles, corresponding to 1.5 × 10^12 ^to 1.5 × 10^5 ^molecules and to a dilution from 10^-3 ^to 10^-10 ^of the competitor fragment, was added to each PCR assay as an internal standard. Relative quantification of specific cDNAs was carried out as previously described [19]. The highest dilution at which the competitor fragment was still detectable for each particular cytokine PCR was arbitrarily defined as 1 unit; the dilution at which cDNA was detectable and the density of the band of the resulting PCR product in agarose gels were used to express the results as multiples of 1 unit. To guarantee reproducibility of the results, PCR was performed in duplicates, which yielded comparable results.

### Assessment of cytokine protein expression

#### Tissue sampling and preparation

In an independent AIA study, samples of knee-joint SM, inguinal LNs, and spleen were obtained from rats killed at six time points: day 0, 6 hours, day 1, day 3, and day 6 of AIA. Cytokine protein analysis was performed on five to six rats per time point. The tissue pieces were snap-frozen in 250 to 1000 μl PBS–EDTA (0.9% NaCl, 30 mM KCl, 70 mM Na_2_HPO_4_, 10 mM KH_2_HPO_4_, 10 mM ethylenediaminetetraacetic acid) containing a proteinase inhibitor cocktail (*Complete*^®^; Roche Diagnostics, Mannheim, Germany), and kept at -70°C until processing. Immediately after thawing, tissue pieces were homogenized using an Ultra Turrax and centrifuged Subsequently the supernatants were divided into aliquots and kept at -70°C.

#### Sandwich ELISA

Concentrations of IL-1β, IL-6, tumor necrosis factor (TNF) α, IFN-γ, IL-4, and IL-10 were determined by sandwich **ELISA **using the monoclonal antibodies MAB501, BAF501 and recombinant rat IL-1β for IL-1β (R&DSystems, Wiesbaden, Germany) or the respective BD OptEIA Sets for all other cytokines (BD Pharmingen, Heidelberg, Germany) in accordance with the manufacturer's recommendations.

Data were normalized to total protein levels as determined using the BCA-Assay (Pierce, Rockville, IL, USA) and expressed as ng cytokine/mg total protein.

### Statistics

The nonparametric Mann–Whitney (*U*) test was applied to analyze differences among groups for all parameters examined. For each cytokine and time point, the levels of mRNA and protein expression were compared with baseline levels (day 0) and with the respective preceding time point. Correlations between cytokine mRNA levels and the severity of joint swelling in individual animals were assessed by means of the Spearman rank correlation test. In both cases, differences were considered statistically significant for *P *≤ 0.05.

## Results

### Clinical parameters

**Figure 1:**
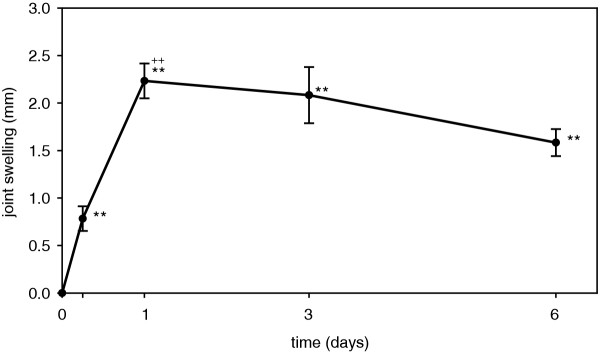
Time course of knee-joint swelling in rats with AIA used to evaluate cytokine mRNA. Values are means (*n *= 4 to 6); vertical bars indicate the standard error of the mean. The disease is characterized by rapid onset of acute inflammation, a peak on day 1, and a transition to chronicity on day 6. **P ≤ 0.01 in comparison with day 0; ^++^*P *≤ 0.01 in comparison with the preceding date. AIA, antigen-induced arthritis.

In both experimental series, arthritis typically developed within 6 hours of intra-articular injection of the arthritogen mBSA and reached a peak on day 1 (Fig. 1); swelling started to decrease on day 3. However, a significantly lower joint swelling in the arthritis series used for determination of protein (data not shown) allowed only a qualitative comparison of mRNA and protein levels in the SM and the other organs.

Generally, the following phases could be distinguished: preclinical (day 0); acute (6 hours to day 3); transition to chronicity (day 6). It should be considered that animals undergoing cytokine mRNA and protein analysis before induction of AIA (day 0) were under the influence of systemic immunization with mBSA (see Materials and methods and the next paragraph for details).

### Cytokine protein levels in the prearthritis phase

For both mRNA and protein, all subsequent data are presented as fold changes in relation to the cytokine expression on day 0 (i.e. after immunization, but before induction of arthritis). Whereas mRNA data are expressed as relative units and are therefore not comparable among different cytokines at any time point, cytokine protein concentrations are expressed as ng/mg total protein and are therefore suitable for comparison among cytokines. Quantitative data for day 0 of AIA are presented in Table 1.

The relative protein expression in the various organs followed nearly identical patterns and quantitatively ranked as follows:

*SM*: IL-1β > TNF-α > IFN-γ > IL-6 > IL-10 > IL-4

*Ing LN*: IL-1β > IL-2 > IFN-γ > TNF-α > IL-10 > IL-6 > IL-4

*Spleen*: IL-1β > IL-2 > TNF-α > IFN-γ > IL-6 > IL-10 > IL-4

In addition, the concentrations of all the cytokines studied showed the highest values in the SM (approximately 2.5 to 340 ng/mg total protein), followed by those in the inguinal LN (approximately 0.02 to 9.8 ng/mg) and spleen (approximately 0.01 to 1.0 ng/mg) on day 0 and also throughout the course of AIA (Table 1, in conjunction with Fig. 3 and Table 3, underlining the predominantly local character of AIA.

### Cytokine mRNA and protein expression in the synovial membrane

**Figure 2:**
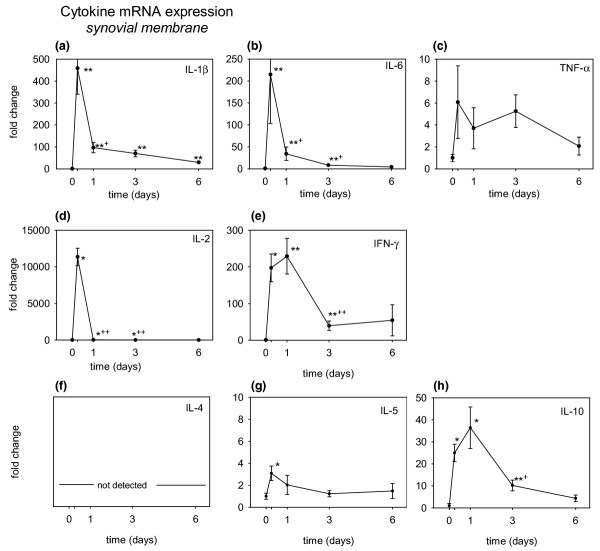
Expression of mRNA of various cytokines in the synovial membrane of rats with AIA. Expression of mRNA was assessed before the induction of antigen-induced arthritis (AIA) (day 0) or afterwards (at 6 hours and on days 1, 3, and 6). Data were originally expressed as arbitrary units (1 unit = highest detectable dilution of the competitor fragment) and then related to the value of day 0 (fold change). Values are means;vertical bars indicate the standard error of the mean (*n *= 4 to 6 rats for each time point). Each determination was performed in duplicate. *P ≤ 0.05, **P ≤ 0.01 in comparison with day 0; ^+^*P *≤ 0.05, ^++^*P *≤ 0.01 in comparison with the preceding date.

**Figure 3:**
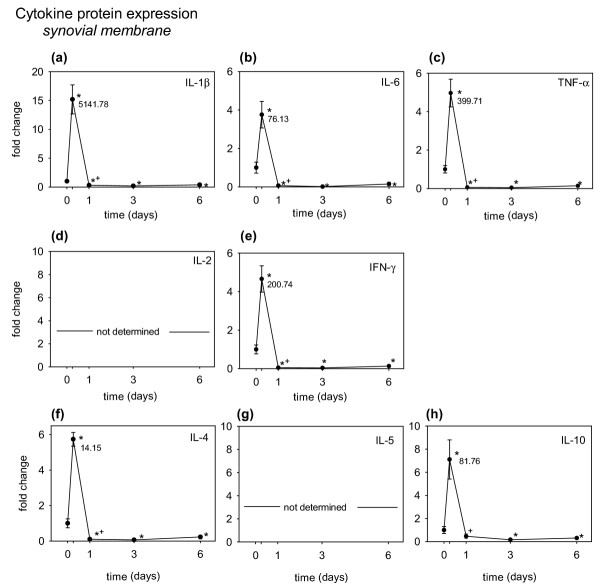
Expression of proteins for various cytokines in the synovial membrane of rats with AIA. Expression of protein was assessed before the induction of antigen-induced arthritis (AIA) (day 0) or afterwards (at 6 hours and on days 1, 3, and 6). Data were originally expressed as ng/mg total protein and then related to values of day 0 (fold change; peak values in ng/mg total protein are indicated for 6 hours). Values are means; vertical bars indicate the standard error of the mean (*n *= 5 to 6 rats for each time point). *P ≤ 0.05 in comparison with day 0; ^+^*P *≤ in comparison with the preceding date.

*IL-1β*. IL-1β mRNA increased sharply by 6 hours after induction of arthritis (Fig. 2a). By days 1 and 3, the expression of this mRNA declined significantly, approaching but still significantly exceeding 'prearthritis' levels by day 6. The mRNA levels correlated positively with the degree of joint swelling in individual animals (*P *= 0.05; Table 2). In general, IL-1β protein levels in the SM matched the mRNA course, with a peak 6 hours after induction (Fig. 3a). In contrast to the mRNA, the protein fell significantly below prearthritis levels already by day 1 and remained at this level until day 6.

*IL-6*. Like IL-1β, IL-6 mRNA levels rose significantly by 6 hours (Fig. 2b), and declined significantly thereafter. On day 6 the levels of this mRNA did not significantly differ from those in the prearthritis phase. IL-6 mRNA levels correlated positively with the degree of joint swelling in individual animals (*P *= 0.03; Table 2). Protein expression followed the course of mRNA expression; that is, after an initial peak at 6 hours (Fig. 3b) IL-6 dropped below prearthritis levels on day 1 and thereafter.

*TNF-α*. TNF-α mRNA levels did not significantly change throughout the disease (although they numerically rose above levels at immunization (Fig. 2c)). However, there was a significant rise of protein at 6 hours after induction (Fig. 3c), followed by a reduction to below prearthritis values on day 1 and thereafter.

*IL-2*. IL-2 mRNA expression underwent a massive elevation at 6 hours, declined significantly on days 1 and 3, and disappeared by day 6 (Fig. 2d). Because of the limited quantity of SM tissue, the protein levels were not determined.

*IFN-γ*. IFN-γ mRNA levels were significantly increased at 6 hours and day 1 and dropped significantly by day 3 (Fig. 2e). The protein increased comcomitantly at 6 hours (Fig. 3e) but had dropped to below prearthritis levels already by day 1.

*IL-4*. Whereas IL-4 mRNA was not detected (Fig. 2f), IL-4 protein was expressed at detectable but low levels. This cytokine increased significantly by 6 hours after induction of AIA (Fig. 3f) and then decreased to below prearthritis values by day 1.

*IL-5*. IL-5 mRNA peaked moderately, but significantly, by 6 hours (Fig. 2g). The protein levels were not determined.

*IL-10*. IL-10 mRNA was notably increased at 6 hours and day 1 and then showed significant decreases on days 3 and 6 (Fig. 2h). The protein increased 6 hours after induction of AIA, followed by a significant decrease on day 1 and a drop to below prearthritis values on days 3 and day 6 (Fig. 3h).

### Cytokine mRNA and protein expression in the inguinal lymph node

**Figure 4:**
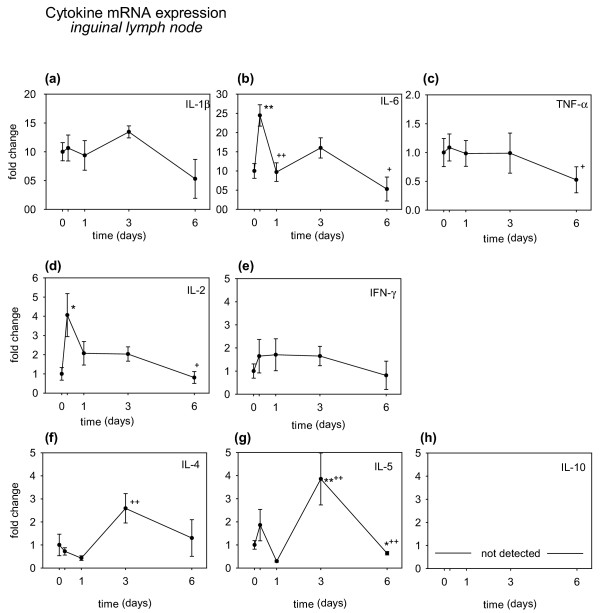
Expression of mRNA for various cytokines in the inguinal lymph nodes of rats with AIA. Time course and other details as in Fig. 2. AIA, antigen-induced arthritis.

*IL-1β*. Neither IL-1β mRNA (Fig. 4a) nor IL-1β protein (Table 3, top) showed major changes throughout the course of AIA (with the exception of a minor, but significant, 2-fold increase of protein by day 6 in comparison with day 0).

*IL-6*. IL-6 mRNA peaked significantly above prearthritis levels by 6 hours after induction of AIA (Fig. 4b), but returned to baseline levels by day 1. On day 3, that is, at the end of the acute peak of AIA, the levels of IL-6 mRNA, although not significantly altered on a group basis, correlated positively with the degree of joint swelling in individual animals (*P *= 0.02; Table 2). No peak of protein concentrations at 6 hours was detected, but protein, too, declined significantly by day 6 in comparison with baseline levels (Table 3, top).

*TNF-α*. TNF-α mRNA maintained immunization levels throughout the acute phase of AIA but dropped significantly by day 6 (Fig. 4c), that is, at the transition to chronicity (Fig. 1); a parallel time course was observed for the protein, though the differences did not reach significance (Table 3, top).

*IL-2*. IL-2 mRNA rose significantly above immunization levels by 6 hours after the induction of AIA and gradually declined to prearthritis levels thereafter (Fig. 4d). The protein showed a similar time course, though the differences did not reach significance (Table 3, top).

*IFN-γ*. The levels of IFN-γ mRNA approximately doubled throughout the acute phase of AIA in comparison with prearthritis levels, though the increase was not statistically significant (Fig. 4e). The protein showed a similar time course, again without reaching significance (Table 3, top).

*IL-4*. IL-4 mRNA expression underwent a significant burst on day 3 of AIA, that is, at the end of the acute phase of the joint disease (Fig. 4f). The protein levels, however, despite a limited increase on day 1, showed no significant changes (Table 3, top).

*IL-5*. IL-5 mRNA expression paralleled that of IL-4 mRNA, also reaching a significant peak on day 3 (Fig. 4g); the expression of IL-4 and IL-5 appeared biphasic, however, as indirectly documented by a drop on day 1 in comparison with prearthritis levels (Fig. 4f,g). Protein levels for IL-5 were not determined.

*IL-10*. IL-10 mRNA was not detected in this organ at any time point of the disease (Fig. 4h), but IL-10 protein was detected at all time points, showing a significant increase on day 1 and a decrease to prearthritis levels on day 6 (Table 3, top).

### Cytokine mRNA and protein expression in the spleen

**Figure 5:**
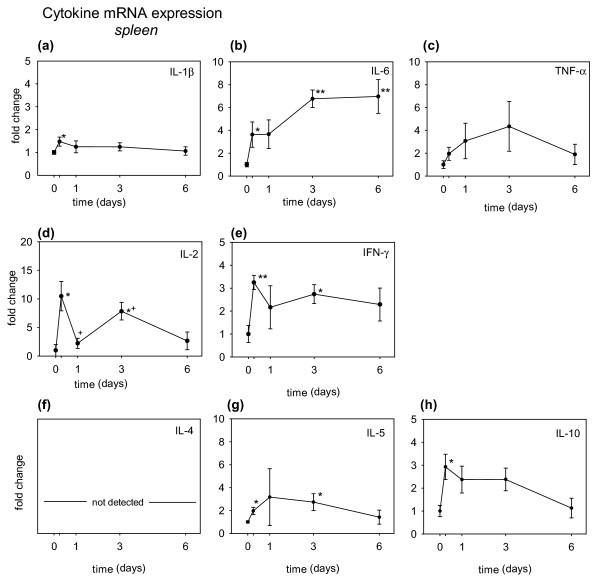
Expression of mRNA for various cytokines in the spleens of rats with AIA. Time course and details as in Fig. 2. AIA, antigen-induced arthritis.

*IL-1β*. IL-1β mRNA levels peaked modestly and only initially, by 6 hours (Fig. 5a). Protein levels were unchanged during the course of arthritis (Table 3, bottom).

*IL-6*. IL-6 underwent a progressive elevation that reached plateau levels between days 3 and 6 of AIA (Fig. 5b), when the clinical signs of synovitis were already decreasing (Fig. 1). The levels of IL-6 mRNA expression correlated positively with the degree of joint swelling (*P *= 0.01; Table 2). Although an elevation of protein levels was not detected between days 0 and 3, a significant increase on day 6 in comparison with day 3 partially reflected the data for the mRNA (Table 3, bottom).

*TNF*α. TNF-α mRNA progressively increased to a peak on day 3; however, the large variability among animals likely impeded statistical significance (Fig. 5c). After an initial significant drop at 6 hours (P = 0.05 in comparison with day 0), the protein levels reached prearthritis levels on day 1 and were slightly, but not significantly, elevated on day 6 (Table 3, bottom).

*IL-2*. IL-2 mRNA showed a biphasic elevation, at 6 hours in correspondence with the beginning of synovitis, and on day 3 (Fig. 5d), coincident with the late acute phase of the disease (Fig. 1). IL-2 protein remained nearly unchanged throughout AIA, with a slight, but significant, increase on day 6 in comparison with day 0 and day 3 (Table 3, bottom).

*IFN-γ*. IFN-γ mRNA underwent a moderate, plateau-like elevation until day 3 of AIA (Fig. 5e), that is, throughout ascending phase and acute peak of AIA (Fig. 1). As with IL-2 protein, a slight, but significant, increase of IFN protein was seen on day 6 in comparison with day 3 (Table 3, bottom).

*IL-4*. IL-4 mRNA was not detected throughout the course of AIA (Fig. 5f). Protein was detectable throughout all phases, though without any significant changes (Table 3, bottom).

*IL-5*. IL-5 mRNA underwent a gradual, moderate rise until day 3 (Fig. 5g); for this cytokine, a negative correlation with the severity of arthritis was observed on day 6 (*P *< 0.001; Table 2), although the group as such showed no significant IL-5 elevation on this date (Fig. 5g). Protein levels for IL-5 were not determined.

*IL-10*. IL-10 mRNA was significantly elevated in the spleen, but only within the acute phase of AIA (6 hours) (Fig. 5h). As with IL-5, IL-10 showed a negative correlation with the degree of joint swelling on day 6 (*P *= 0.04; Table 2). There were no significant changes of IL-10 protein levels throughout the course of AIA (Table 3, bottom).

There was no significant correlation between the clinical time course and the protein levels for any cytokine in any organ at any time point.

## Discussion

The present study documents that, in AIA, elevation of IL-1β in the SM as well as elevations of IL-6 mRNA in SM, inguinal LN, and spleen correlate positively with the disease severity and that the rise of Th1-like cytokines in the SM is massive in this model, but that this rise clearly overlaps with early Th2-like responses, as has also been shown by immunohistochemistry in the respective mouse model [21].

### Local compartment

At early stages of AIA, the elevation of mRNA for IL-1β and IL-6 is very prominent at the primary site of pathology (SM 450- to 200-fold >> LN and spleen 1.5- to 10-fold; Figs 2, 4, and 5); this is very consistent with the prominently local character of AIA, induced by direct intra-articular injection of the arthritogen mBSA [15]. The levels of gene activation for these cytokines in the SM correlate positively with the clinical severity of AIA, as has also been reported for IL-1β and IL-6 protein in murine or rabbit AIA [5,22]. In the systemic rat adjuvant arthritis, in contrast, IL-1β is far more elevated in the LN than in the SM [19], in line with the different mode of induction of the disease, which favors spread of the arthritogen to the regional LNs [23]. Both the pathology and the sequence of macrophage immigration in the inflamed SM are well characterized in AIA [[24,25], and our own observation]. However, the early profiles of IL-1β and IL-6 mRNA do not match these kinetics. Similarly, there is no obvious relationship with the distribution of macrophage subpopulations, as identified by ED1, ED2, or ED3 markers [26]. It must be considered that the normal SM in the rat contains a number of resident macrophages [27] that, once activated, could be responsible for the early production of monokines in AIA; these monokines may initiate the inflammatory response and promote further cell immigration [28].

AIA synovitis is accompanied by a nonsignificant 6-fold local elevation of TNF-α message (Fig. 2c), but a significantly increased protein production (5-fold; peak level approximately 400 ng/mg total protein; Fig. 3c). In comparison with other cytokines such as IL-6 (200-fold increase of mRNA; 4-fold increase of protein; peak level approximately 80 ng/mg total protein), TNF-α reached higher protein levels despite lower fold changes of mRNA, possibly because of a more efficient translation mechanism. Such discrepancies between mRNA and protein expression have also been found in another study, using streptococcal-cell-wall-induced arthritis [29]: a 100,000-fold mRNA increase for IL-6 resulted in only a 1000-fold protein elevation, whereas the increases for TNF-α were 3.5-fold and 2-fold for mRNA and protein, respectively.

In the SM, there were significant elevations of both IFN-γ and IL-2 mRNA in the acute phase (Figs 2d,e; also confirmed for IFN-γ protein in Fig. 3e), thus indicating a complete Th1-like response at a local level. The burst of these cytokines is early and short-lasting, representing therefore a marker of very acute disease, and supporting the concept that anti-IL-2- or anti-IFN-γ treatments are of potential therapeutic use if performed at the outbreak of disease [30,31]. The marked elevation of both IFN-γ and IL-2 mRNA at the primary site of pathology, in contrast to the modest elevation of these lymphokines at a regional and systemic level, is consistent with the pre-eminently local character of AIA. Notably, this profile is practically the opposite to that of systemic adjuvant arthritis, in which early IFN-γ mRNA elevation is prominent in the regional LN draining the injection site of the arthritogen, but very modest in the SM [19]. Strong Th1-like responses, therefore, appear limited to the site of antigen injection, occurring only upon massive exposure of the local immune system to the antigen. Whether the early IL-2 mRNA peak in the SM of AIA rats also contributes to the development of regulatory T cells remains to be determined [32].

Overlapping the Th1-like response, there were significant elevations of the Th2 cytokines IL-4 (protein only; Fig. 3f), IL-5 (mRNA; Fig. 2g; protein not determined), and IL-10 (Figs 2h and 3h). Of particular interest, IL-10 in the SM peaks and then progressively declines until chronicity ensues (Figs 2h and 3h). The role of IL-10 in arthritis clearly seems a protective one, as indicated by studies on the amelioration of collagen-induced arthritis by administration of IL-10 or its augmentation in IL-10 knockout mice [33,34]. IL-10 is a Th2-like cytokine produced not only by Th1 and Th2 cells, but also (and perhaps predominantly) by macrophages, probably as an autocrine factor of immune regulation (reviewed in [8]). The very early rise of IL-10 in the SM supports this view, because in this organ it coincides with massive macrophage activation (Figs 2 and 3), which is probably due to locally injected, arthritogenic mBSA.

The acute rise of IL-4 in the SM was detected only with regard to protein, possibly due to the extremely sensitive ELISA-Kit (detection limit 0.1 pg/ml) capable of detecting very low amounts of this cytokine in the SM (2.5 ng/mg total protein). The early expression of IL-4 seems to be important to counteract the dramatic inflammatory response in acute arthritis, as is shown by a protective effect of IL-4 administration in the induction phase of CIA [35]. However, lower acute responses but disease-promoting effects in the chronic phase of CIA have been reported in IL-4^-/- ^mice [36], showing a phase-dependent, dual role of this cytokine.

The acute rise of IL-5 mRNA in the SM could be IL-4-dependent, based on the fact that IL-4 is a driving force in many Th2-like responses [8]. Of note, early IL-5 rises have also been observed in adjuvant arthritis [19], and, more importantly, in Biozzi mice susceptible (but not in mice resistant) to collagen-induced arthritis [37]. Timed IL-5- or anti-IL-5 treatments are therefore needed to address the proinflammatory or anti-inflammatory role of IL-5 in the acute phase of AIA.

### Systemic compartments

In spite of its prominently local character, AIA is accompanied by a peak of IL-6 mRNA in the inguinal LN at 6 hours (Fig. 4b), and an elevation of this mRNA in the spleen (6 hours, day 3, and day 6; Fig. 5b), in the latter case significantly correlated with the severity of disease (Table 2). This rise is maintained throughout the chronic phase, similarly to IL-6 protein in the serum of AIA [38] and adjuvant arthritis rats [39], and in analogy to findings in the synovial fluid of RA patients [40]. Whereas the acute rise of LN/spleen IL-6 is consistent with the acute-phase response typical of early AIA [41], the contribution of IL-6 to chronicity remains uncertain [42,43], perhaps because it can be produced not only by macrophages but also by Th2 cells [8,39].

TNF-α mRNA can be clearly documented in the regional LN and spleen (Figs 4c and 5c), in temporal coincidence with the severity of the joint swelling (Fig. 1), though with high variability from animal to animal, resulting in a lack of statistical significance. In AIA, therefore, the role of systemic TNF-α appears marginal, at least in relation to other cytokines, as has also been shown by the fact that successful anti-CD4 therapy reduced IL-6, but not TNF-α levels in local and systemic compartments [5]. This is at odds with more systemic models of arthritis, such as collagen-induced and adjuvant arthritis [19,44], in which there is highly significant TNF-α elevation in LN and/or spleen. Spleen TNF-α, in particular, is significantly correlated with the severity of the wasting syndrome in adjuvant arthritis [45]. The modesty of spleen TNF-α changes in AIA (Fig. 5c), therefore, is consistent with the lack of a wasting syndrome in this model [17].

Both inguinal LN and spleen showed an increase of IL-2 mRNA in the acute phase of AIA (Figs 4d and 5d), similar to a significant rise of IFN-γ in the spleen (Fig. 5e). This is clearly in line with the concept of Th1 dominance in AIA, as is also emphasized by the reduced levels of these Th1 cytokines in spleen and LN upon successful anti-CD4 treatment of AIA in mice [4]. The second elevation of IL-2 and IFN-γ in the spleen just before the transition to chronicity is similar to the situation in rat adjuvant arthritis [19], and suggests that recruitment of fresh, possibly disease-controlling, regulatory T cells [17,32] may have a systemic component even in the predominantly local AIA.

Although IL-4 mRNA underwent no changes in the spleen, it rose significantly in the inguinal LN on day 3 (Fig. 4f). Furthermore, there was a significant elevation of IL-5 mRNA in spleen and inguinal LN on day 3. Both findings are consistent with the transition to a regulatory phase of T-cell function in anticipation of chronicity. This time point may therefore represent the turning point of the disease, when LN-generated Th2-like responses may gradually replace Th1-like processes, or, according to the present data, when the Th1/Th2-like balance shifts in favor of Th2-like patterns [46]. The lack of significant elevation of Th2-like cytokines in lymphoid organs at the protein level may be due to the relatively weak clinical arthritis in this experimental series.

### Overlap of Th1-like and Th2-like responses

Besides somewhat obvious Th2-like elevations in advance of chronicity (see above), in line with the expected regulatory properties of this group of cytokines [8,47,48], clear Th2-like peaks markedly overlap with the initial Th1-like surge. In the LN, the early Th2-like rise may include not only IL-5 and IL-10, but also IL-6, inasmuch as this cytokine can be produced by Th2-like cells [8] and not only by macrophages, which are modestly activated in this organ (Fig. 4a–c). These findings document that a sharp Th1/Th2 division, valid for some other systems [8], does not automatically apply to *in vivo *models of autoimmunity [10], including other models of arthritis [19,36]. The biological relevance of the early Th2-like rise in the SM and at systemic sites remains however unclear, that is, whether it contributes to inflammation or rather represents an attempt to limit the acute inflammatory insult [8].

The Th1-like/Th2-like responses overlap with some degree of anatomical segregation. While in the SM and spleen the expression of mRNA for IL-2 and IFN-γ overlaps with that of IL-5 and IL-10, the inguinal LN shows an overlap of mRNA for IL-2 with IL-4 and IL-5. A clear anatomical segregation of Th1-like/Th2-like responses, although in different patterns, is seen also in rat adjuvant arthritis [19]. The anatomic location of these responses varies, however, in the two models; the knee monoarticular arthritis in AIA seems to require a more regionally confined crosstalk with the inguinal LN, whereas the systemic adjuvant-induced polysynovitis requires a much stronger involvement of the spleen, the organ in which potentially regulatory T-cell cytokine responses appear generated.

## Conclusion

The present study documents that the course of AIA is characterized by organ-specific overlaps of Th1-like and Th2-like responses. Activation of synovial macrophages and T cells is prominent in this prevalently local model of arthritis, although regional and systemic factors may also contribute to the disease processes. The cytokine patterns share some features with systemic adjuvant arthritis, although there are several clear differences, conceivably imputable to pathogenetic differences between the two models.

## Abbreviations

AIA = antigen-induced arthritis; ELISA = enzyme-linked immunosorbent assay; IFN = interferon; IL = interleukin; LN = lymph node; mBSA = methylated bovine serum albumin; PBS = phosphate-buffered saline; RA = rheumatoid arthritis; RT-PCR = reverse transcriptase polymerase chain reaction; SM = synovial membrane; Th = T-helper; TNF = tumor necrosis factor.

## Competing interests

The author(s) declare that they have no competing interests.

## Authors' contributions

DP performed the assessment and analysis of the protein data and participated in writing the manuscript. AS, EB, and CBSW assessed and analyzed the mRNA data. EPK critically read and edited the manuscript. FE and RB participated in the design and coordination of the study, including the animal experiments. RWK contributed to the design of the study, including the animal experiments, and participated in the layout, writing, and finalization of the manuscript. All authors read and approved the final manuscript.

## Figures and Tables

**Table 1:** Cytokine protein per total protein (ng/mg) on day 0 of antigen-induced arthritis in rats

Cytokine	Synovial membrane	Inguinal lymph node	Spleen
IL-1β	338.60 (90.78)	9.81 (2.61)	0.983 (0.117)
IL-6	20.26 (5.77)	0.10 (0.03)	0.015 (0.006)
TNF-α	80.46 (15.48)	0.71 (0.19)	0.041 (0.004)
IL-2	n.d.	1.73 (0.54)	0.054 (0.012)
IFN-γ	43.11 (9.83)	0.74 (0.21)	0.028 (0.004)
IL-4	2.47 (0.62)	0.02 (0.01)	0.007 (0.001)
IL-10	11.51 (3.49)	0.56 (0.11)	0.011 (0.001)

Values are means (standard error of the mean). n.d., not determined.

**Table 2:** Correlation between mRNA expression and severity of knee joint swelling in rats with AIA

Cytokine	Day(s)	Correlation^a^	*P*^a^	ρ^a^	*n*
*Synovial membrane*					

IL-1β	0–6	+	0.05	0.527	25
IL-6	0–6	+	0.03	0.405	28

*Inguinal lymph node*					

IL-6	3	+	0.02	0.886	6

*Spleen*					

IL-6	0–6	+	0.01	0.511	25
IL-5	6	-	<0.001	1.000	4
IL-10	6	-	0.04	0.829	6

^a^Spearman rank correlation. +, positive; -, negative; AIA, antigen-induced arthritis.

**Table 3:** Changes (fold change relative to day 0) in cytokine protein concentrations during rat AIA

Time point	IL-1β	IL-6	TNF-α	IL-2	IFN-γ	IL-4	IL-10
*Inguinal lymph node*							

Day 0	1.00 (0.27)	1.00 (0.26)	1.00 (0.26)	1.00 (0.31)	1.00 (0.28)	1.00 (0.25)	1.00 (0.20)
6 hours	1.67 (0.60)	0.98 (0.38)	1.93 (0.78)	1.06 (0.31)	0.98 (0.25)	1.38 (0.55)	1.60 (0.32)
Day 1	1.74 (0.58)	0.59 (0.42)	2.53 (0.78)	1.31 (0.45)	1.14 (0.34)	1.86 (0.72)	2.18* (0.49)
Day 3	1.24 (0.29)	0.40 (0.21)	1.38 (0.28)	0.66 (0.12)	0.55 (0.11)	0.53 (0.28)	1.41 (0.24)
Day 6	1.86* (0.41	0.10* (0.10)	1.87 (0.38)	0.69 (0.14)	0.81 (0.12)	0.91 (0.27)	0.89 (0.17)

*Spleen*							

Day 0	1.00 (0.12)	1.00 (0.41)	1.00 (0.10)	1.00 (0.23)	1.00 (0.13)	1.00 (0.12)	1.00 (0.05)
6 hours	0.75 (0.09)	0.32 (0.14)	0.61* (0.04)	0.70 (0.07)	0.78 (0.04)	0.77 (0.04)	0.94 (0.06)
Day 1	0.72 (0.11)	0.41 (0.17)	0.95 (0.13)	0.84 (0.16)	0.81 (0.07)	0.80 (0.08)	0.87 (0.08)
Day 3	0.81 (0.06)	0.24 (0.14)	1.07 (0.08)	0.70 (0.13)	0.71 (0.06)	0.85 (0.06)	0.90 (0.06)
Day 6	0.67 (0.06)	0.82*^+ ^(0.07)	1.36 (0.21)	1.10*^+ ^(0.13)	0.91^+ ^(0.06)	0.96 (0.10)	0.99 (0.07)

Values (fold changes) are means (standard error of the mean) (*n *= 5 to 6 rats for each time point). AIA, antigen-induced arthritis. *P ≤ 0.05 in comparison with day 0; ^+^*P *≤ 0.05 in comparison with the preceding date.
